# Higher Levels of Protein Palmitoylation in the Frontal Cortex across Aging Were Associated with Reference Memory and Executive Function Declines

**DOI:** 10.1523/ENEURO.0310-18.2019

**Published:** 2019-02-07

**Authors:** Daniel R. Zamzow, Valerie Elias, Varinia A. Acosta, Emily Escobedo, Kathy R. Magnusson

**Affiliations:** 1 University of Wisconsin-Whitewater, Janesville, Wisconsin 53546; 2 Department of Biomedical Sciences, College of Veterinary Medicine, Oregon State University, Corvallis, Oregon 97331; 3 Linus Pauling Institute, Oregon State University, Corvallis, Oregon 97331

**Keywords:** aging, memory, Morris water maze, NMDA receptor, palmitoylation, prefrontal cortex

## Abstract

Cognitive decline with aging is often due to altered levels of protein expression. The NMDA receptor (NMDAR) and the complex of proteins surrounding the receptor are susceptible to age-related changes in expression. In the frontal cortex of aged mice, there is a significant loss of expression of the GluN2B subunit of the NMDAR, an increase in Fyn expression, and no change in PSD-95. Studies have also found that, in the frontal cortex, phosphorylation of GluN2B subunits and palmitoylation of GluN2 subunits and NMDAR complex proteins are affected by age. In this study, we examined some of the factors that may lead to the differences in the palmitoylation levels of NMDAR complex proteins in the frontal cortex of aged animals. The Morris water maze was used to test spatial learning in 3- and 24-month-old mice. The acyl–biotinyl exchange method was used to precipitate palmitoylated proteins from the frontal cortices and hippocampi of the mice. Additionally, brain lysates from old and young mice were probed for the expression of fatty acid transporter proteins. An age-related increase of palmitoylated GluN2A, GluN2B, Fyn, PSD-95, and APT1 (acyl protein thioesterase 1) in the frontal cortex was associated with poorer reference memory and/or executive functions. These data suggest that there may be a perturbation in the palmitoylation cycle in the frontal cortex of aged mice that contributes to age-related cognitive declines.

## Significance Statement

Cognitive decline due to aging significantly impacts the quality of life of those affected, as well as their families. Although data have shown that there is not a dramatic loss of neurons in the aged brain, the expression of some proteins essential for memory dwindle with advanced age. However, other proteins remain constant or increase with age. Post-translational modification of proteins via palmitoylation significantly alters the function of signaling molecules in the brain. We found an age-related increase in the percentage of NMDA receptor-associated proteins that were palmitoylated in the frontal cortex of mice, which were associated with age-related deficits in memory and executive functions. This finding may point to a previously unexplored mechanism of cognitive decline in older individuals.

## Introduction

Cognitive decline due to aging is quite evident by 50 years of age in humans and many may feel the effects of memory loss by 40 years of age (Scherr et al., 1988; Singh-Manoux et al., 2012). Both long-term (reference) and short-term memory show declines with age in humans (Scherr et al., 1988; Baddeley et al., 1991) and in mice (Magnusson et al., 2003). Spatial memory involves the ability to navigate through one’s surroundings and also shows signs of deterioration with age (Gallagher et al., 1993). Aspects of age-related damage of spatial memory are evident in many mammalian species, and, as such, tools like the Morris water maze can be used with humans as well as rodents (Gallagher et al., 2011; Zhong et al., 2017).

One of the neural receptors that is key to performing memory tasks is the NMDA receptor (NMDAR; Morris et al., 1986). Subunits of the NMDAR show significant age-related declines of protein expression in the hippocampus and frontal cortex (Magnusson et al., 2002, 2007, 2010; Das and Magnusson, 2011). There is a significant loss of protein expression of the GluN2B subunit in most cellular fractions in the hippocampus of aged mice; however, greater effects due to age are found in the synaptic membrane in the frontal cortex and are related to reference memory declines (Magnusson et al., 2007; Zhao et al., 2009; Zamzow et al., 2016).

The NMDAR interacts with many different proteins (Husi et al., 2000). NMDARs are concentrated and held at synapses by PSD-95 (Roche et al., 2001; Xu, 2011). The Src kinase, Fyn, phosphorylates tyrosines 1336 (p1336) and 1472 (p1472) on GluN2B, which influences localization of GluN2B-containing receptors within the synaptic terminal and long-term potentiation (Nakazawa et al., 2001; Goebel-Goody et al., 2009). Post-translational modification influences the actions of all the NMDAR complex proteins. Protein palmitoylation is a post-translational modification that is an important regulator of synaptic plasticity (Fukata and Fukata, 2010). Palmitoylation of GluN2A and GluN2B subunits, as well as PSD-95 and Fyn, clusters the proteins on detergent-resistant postsynaptic membranes and influences phosphorylation events that govern trafficking of the proteins (El-Husseini et al., 2002; Hayashi et al., 2009; Noritake et al., 2009; Sato et al., 2009). Despite the essential role of palmitoylation in synaptic plasticity (Fukata and Fukata, 2010), age-related increases in palmitoylation of amyloid precursor protein may also be responsible for the pathogenesis of Alzheimer’s disease (Bhattacharyya et al., 2013).

The protein palmitoylation cycle is governed by protein acyltransferases (PATs) that attach palmitates to free sulfhydryls on proteins via a labile thioester bond, and protein thioesterases (APTs) that remove the palmitate (Baekkeskov and Kanaani, 2009; Conibear and Davis, 2010; Yokoi et al., 2016). The substrate for protein palmitoylation is palmitoyl-CoA (Greaves and Chamberlain, 2011), which is formed by esterifying palmitate that has crossed the blood–brain barrier. Palmitate can be transported across the blood–brain barrier by the fatty acid transport proteins (FATPs), FATP 1, and fatty acid translocase (i.e., CD36; Mitchell et al., 2011). Palmitate is esterified in the brain by long-chain acyl-CoA synthetase 6 (ACSL6; Van Horn et al., 2005). Unfortunately, very little is known about how these proteins are affected by the aging process.

A recent study (Zamzow et al., 2014) found that the number of GluN subunits, Fyn, and PSD-95 proteins that are palmitoylated increases with age in the frontal cortex, but not the hippocampus of mice. Another study (Zamzow et al., 2016), which divided older mice into good and bad reference memory learners based on average place trial performance in the Morris water maze, showed an increase in p1472 on GluN2B in the synaptic membranes from frontal cortex of older good reference learners only, but enhanced Fyn in the same fraction in the poor older learners. It is not known whether a disturbed palmitoylation cycle can explain altered localization and phosphorylation of proteins in the frontal cortices of old mice. Also unknown are the exact mechanisms driving the perturbations in protein palmitoylation, phosphorylation, and localizations in the frontal cortices of old mice. In the current study, we assessed the spatial memory of C57BL/6 mice of two different ages, with older mice divided into good and bad reference learners; analyzed protein palmitoylation; quantified levels of fatty acid transport proteins; and examined palmitoylation and subcellular localization of acyl protein thioesterase 1 (APT1). We found no links to reference learning status within old mice, but correlational analysis across aging showed links between protein palmitoylation and other behavioral measures, particularly performance in a delayed matching-to-place task.

## Materials and Methods

### Animals

A total of 35 male C57BL/6 mice from two age groups (3–5 and 24 months of age) were used for this study. Mice in the older age group were obtained from National Institute on Aging of the National Institutes of Health (NIH). Young mice were purchased from The Jackson Laboratory, which stocks the NIH colony. Mice were fed *ad libitum* and housed with a 12 h light/dark cycle. Thirteen mice [7 young (5 months old), 6 old] in Study 1 were fed the defined AIN-93G diet. Subfractionated tissue from a previous study (Zamzow et al., 2016) was also used in Study 2, in which 24 mice [12 young (3 months old), 12 old] were fed a standard chow diet (LabDiet). After the behavioral testing, all animals were killed by exposure to CO_2_ and decapitated. The brains were harvested, frozen in dry ice, and stored at −80°C.

### Behavioral testing

Spatial reference memory, cognitive flexibility, and associative memory (cued control task) were tested, with the use of the Morris water maze, as previously described (Das et al., 2012), in both studies, but testing was reduced in Study 2. Briefly, for the first 2 d, all mice were acclimated to the water maze, followed by 2 d (Study 2) or 3 d (Study 1) of testing for spatial reference memory, 1 d of reversal training to test cognitive flexibility, 7 d of delayed matching-to-place testing (Study 1 only), and 1 d of associative memory testing (cued control task). Reference memory testing consisted of eight place trials per day and one probe trial at the end of each day. A naive probe trial was performed at the beginning of the first day of memory testing. The platform was kept in the same quadrant for each place trial. Place trials consisted of a maximum of 60 s in the water searching for the platform, 30 s on the platform and 2 min of cage rest. If a mouse failed to find the platform within the designated 60 s swim time, it was led to the platform by the experimenter. Probe trials were performed to assess the ability of the animal to show a bias for the platform location. During the probe trial, the platform was removed, and the mouse was allowed to search in the water for 30 s. After 2–3 d of place and probe trials, a reversal task was performed to assess cognitive flexibility. The platform was placed in the opposite quadrant in the tank, and place and probe trials were performed that were similar to 1 d of the reference memory task.

The Study 1 mice were also tested in a spatial delayed matching-to-place task, as previously described (Das and Magnusson, 2011). The task consisted of two sessions per day for 7 d. The platform positions were changed between each session. Each session consisted of four trials. The first trial was a naive trial in which the mouse was allowed to search for the platform position for a maximum of 60 s, after which the mouse was allowed to remain on the platform for 30 s, followed by cage rest for 10 min (delay period). In the second trial, the mouse was placed in the water at a different entry point from the naive trial and allowed to search for the platform for a maximum of 60 s. The mouse was again allowed to stay on the platform for 30 s and allowed to rest in the cage for 2 min. The mouse was placed into the water two more times at two different entry points and was allowed to find the platform for 60 s. They spent 30 s on the platform and rested in the cage for 2 min between trials. Mice were then placed into their cages until the next session, which started at least 3 h from the beginning of the first session. If the mouse failed to find the platform within the designated 60 s for any of the trials, it was led to the platform by the experimenter. The entry points within one session were randomly assigned for each trial. Delayed matching-to-place task performance was assessed between naive and delay trials.

Cued trials were designed to test motivation, visual acuity, and physical ability for the task. The mice performed six cued trials. The positions of entry and the platform positions varied between trials. The platform was kept submerged, but was marked by a 20.3 cm support with a flag. The mice were allowed to search for the platform for 60 s. The movements of the animal in the water maze were tracked and analyzed with the SMART tracking system (San Diego Instruments).

### Tissue processing for acyl–biotinyl exchange

To quantify levels of protein palmitoylation, proteins were subjected to the acyl–biotinyl exchange method (ABE), as previously described (Wan et al., 2007). The frontal cortices (rostral 4 mm of cortex, with olfactory bulbs and caudate nuclei removed) and hippocampi (cortex isolated from brainstem and hippocampus flipped off the cortex and separated along the white matter) from Study 1 mice (five young, six old) were homogenized on ice in a Dounce homogenizer with 500 μl of buffer LB [50 mm Tris-HCl, pH 7.4, 150 mm NaCl, 5 mm EDTA, protease inhibitor cocktail (Sigma-Aldrich)] and 10 mm *N*-ethylmaleimide (NEM). Homogenization involved 12 strokes from each of two pestles of increasing sizes in homogenization buffer. Aliquots were taken from each sample to analyze total fatty acid transporter proteins. After homogenization, Triton X-100 was added to a final concentration of 1.7%, and the mixture were incubated with rotation at 4°C for 2 h. Excess NEM was stripped, and proteins were precipitated with three sequential chloroform/methanol (1:3, v/v) precipitations. Precipitated proteins were solubilized in 300 μl of 4SB (50 mm Tris-HCl, pH 7.4, 150 mm NaCl, 5 mm EDTA, 4% SDS), and diluted with 1.2 ml of +HA buffer (0.7 m hydroxylamine, pH 7.4, 0.4 mm *N*-[6-(biotinamido)hexyl]-3´-(2´-pyridyldithio)propionamide (HPDP-biotin; Pierce), 0.2% Triton X-100, 150 mm NaCl, protease inhibitor cocktail) or 1.2 ml of −HA buffer (50 mm Tris-HCl, pH 7.4, 0.4 mm HPDP-biotin, 150 mm NaCl, 0.2% Triton X-100). The mixtures were incubated with rotation at room temperature for 2 h, followed by three sequential chloroform/methanol (1:3, v/v) precipitations. Precipitated proteins were solubilized in 150 μl of 2SB buffer (50 mm Tris-HCl, pH 7.4, 2% SDS, 5 mm EDTA, 150 mm NaCl, protease inhibitor cocktail) and diluted in 2.8 ml of buffer LB + 0.2% Triton X-100. Proteins were precipitated from the mixture by incubation with 60 μl of streptavidin-agarose (Pierce) for 2 h at room temperature with rotation. Beads were pelleted and washed three times in buffer LB, and proteins were eluted by boiling the beads in 150 μl of buffer LB + 10% β-mercaptoethanol.

### Tissue processing for cell subfractionation

Biochemical fractionation, following dissection of frontal cortex and hippocampus as described above, was performed on 23 mice (11 young, 12 old; Study 2) as previously described (Dunah and Standaert, 2001; Fan et al., 2012), with a few modifications (Zamzow et al., 2016). Briefly, tissue was homogenized on ice with a Dounce homogenizer in Tris-EDTA buffer (10 mm Tris-HCl, pH 7.4, 1 mm EDTA, 1 mm EGTA) plus 320 mm sucrose and protease inhibitor cocktail (Sigma-Aldrich). Homogenate was centrifuged at 4°C 1000 × *g* for 10 min and the resulting pellet (P1) was discarded. The supernatant (S1) was centrifuged at 4°C 12,000 × *g* for 20 min in an Eppendorf centrifuge to produce the crude synaptosome pellet (P2) and the supernatant cytosolic and microsomal fraction (S2). The P2 fraction was then solubilized in Triton X-100 (Sigma-Aldrich) and fractionated as previously described (Milnerwood et al., 2010). The P2 pellet was resuspended with 300 µl of Triton buffer (10 mm Tris-HCl, 100 mm NaCl, 0.5% Triton, pH 7.2) and rotated slowly (15 min, 4°C), followed by centrifugation (12,000 × *g*, 20 min, 4°C). The supernatant (Triton-soluble fraction) containing non-PSD membranes was retained. The P2 pellet was resuspended in 150 µl of SDS buffer (10 mm Tris-HCl, 150 mm NaCl, 1% Triton X-100, 1% deoxycholic acid, 1% SDS, 1 mm DTT, pH 7.5), followed by gentle rotation (1 h, 4°C) and centrifugation (10,000 × *g*, 15 min, 4°C). The pellet was discarded and the supernatant (Triton-insoluble PSD fraction) retained. Microsomal and cytosolic (S2), PSD (TxP), and non-PSD (TxS) samples were stored at −80°C.

### Western blot

SDS-PAGE (10%) was used for Western blotting as described previously (Zhao et al., 2009). Each gel contained four different loads (2, 4, 8, and 16 μg/well) of standards, obtained from homogenate prepared by combining caudal cortices from all young animals. Protein samples from representatives of each different reference memory status group were loaded on each gel and analyzed in triplicate. Proteins were transferred to PVDF membranes, blocked in Odyssey blocking buffer (LI-COR)/Tris-buffered saline (TBS; 1:1, v/v) and incubated at 4°C in primary antibodies, membranes rinsed three times with TBS-T, and incubated in fluorescence-based secondary antibody. Bands were visualized by scanning in the LI-COR Odyssey Imager. See Table 1 for antibody dilutions and sources.

**Table 1.** Antibody dilutions, sources and catalog numbers used for Western blots

	Dilution	Source		Dilution	Source
GluN2B	1:1000	Millipore 06-600	CD36	1:250	Santa Cruz Biotechnology sc-9154
PSD-95	1:1000	Thermo Fisher Scientific MA1-046	ACSL6	1:250	Santa Cruz Biotechnology sc-134498
APT1	1:1000	Abcam ab91606	FATP1	1:250	Santa Cruz Biotechnology sc-25541
GluN2A	1:250	Santa Cruz Biotechnology sc-136004	Actin	1:250	Santa Cruz Biotechnology sc-1616
Fyn	1:250	Santa Cruz Biotechnology sc-271294	Flotillin	1:250	Santa Cruz Biotechnology sc-25506
			Secondary	1:5000	Rockland

### Data analysis

**Figure 1.**
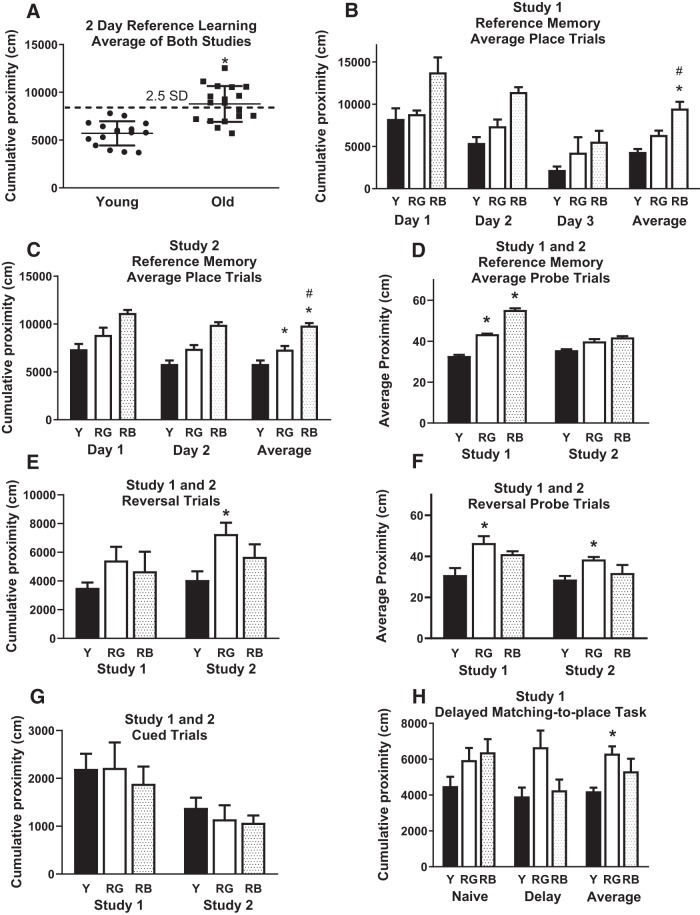
Behavioral data. Aged mice from combined Study 1 and 2 had significantly poorer reference memory than young mice. ***A***, Mice were separated by reference memory status (dotted line). ***B***, ***D–F***, For Study 1, significant differences were seen in reference memory (***B***), probe trials (***D***), and reversal probe trials (***F***), but not reversal place trials (***E***). ***C***, ***E***, ***F***, Significant differences were evident in Study 2 for reference (***C***), reversal (***E***), and reversal probe trials (***F***). ***H***, For Study 1, RG had significantly higher cumulative proximities than young overall in the naive and delay trials in the delayed matching-to-place task. ***G***, No significant difference was noted in cued trials. *p < 0.05 for difference from young. #*p* < 0.05 for difference from RG. *N* = 3–5. Data are the mean ± SEM. Y, Young. RG, reference memory good. RB, reference memory bad.

Data for behavioral testing were analyzed as previously described (Das et al., 2012). Cumulative proximity measures, which reflect search distance from the platform, were used for the place, reversal, delayed matching-to-place task, and cued trials, and average proximity measures were used for probe trials (Gallagher et al., 1993). The average reference memory acquisition performance over 2 d from individuals in the two studies were analyzed together, and the old mice were divided into the categories of “Reference memory-Good” (RG) and “Reference memory-Bad” (RB) learners as previously described (Lee et al., 1994; Rowe et al., 2007; Yetimler et al., 2012) with some modification. The criterion for “reference-bad” old learners was established by selecting old mice with average reference scores that were 2.5 SDs above the mean of the young mice, while “reference-good” old learners were within the same 2.5 SD window (Fig. 1*A*). This division was near the mean and median of the old mice. A total of three young mice (two from Study 1 and one from Study 2) were also identified as RB learners, using the same criteria. They were removed from further analysis and palmitoylation assessment because of the low N for young RB within each study. Protein blots were analyzed using LI-COR Odyssey software. Integrated intensity measures were obtained using median background subtraction method. A standard curve was obtained using a linear fit with Excel (Microsoft) from integrated intensity values for known loads of caudal cortex. Sample values were interpolated from the standard curve as caudal cortex equivalents. Each protein was normalized to β-actin within each sample of total protein. Because the relative percentage of flotillin molecules that are palmitoylated does not change with age (Bhattacharyya et al., 2013), all proteins precipitated through ABE were normalized to flotillin. Behavioral trials were analyzed by repeated-measures ANOVA [Reference Memory Status (i.e., young vs RG vs RB) × Day or Trial]. Averaged behavioral data and protein expressions were assessed with ANOVA for Reference Memory Status, followed by Fisher’s protected least significant difference (PLSD) using StatView software (SAS Institute). GraphPad Prism software was used to calculate Pearson’s correlational coefficients between protein palmitoylation levels and memory performance, and Student’s *t* test was used to validate the continuity of flotillin palmitoylation across ages.

## Results

### Behavioral analysis

There was an overall effect of age when data for 2 d reference memory place trials were combined for Study 1 and Study 2 mice (*F*_(2,31)_ = 45.149, *p* < 0.0001; Fig. 1*A*; *a*; Table 2). Older mice were divided into good and bad reference memory learners, based on a cutoff of 2.5 SDs from the mean of the young from both studies (Fig. 1*A*).

**Table 2.** Data structure table

	Data structure	Type of test	*Post hoc* power
a	Normal	ANOVA	*p* < 0.0001
b	Normal	Repeated-measures ANOVA	*p* = 0.007
c	Normal	Repeated-measures ANOVA	*p* = 0.001
d	Normal	Repeated-measures ANOVA	*p* = 0.0008
e	Normal	Repeated-measures ANOVA	*p* = 0.28
f	Normal	ANOVA	*p* = 0.0228
g	Normal	Repeated-measures ANOVA	*p* = 0.0025
h	Normal	Repeated-measures ANOVA	*p* = 0.0695
i	Normal	Repeated-measures ANOVA	*p* = 0.82
j	Normal	Repeated-measures ANOVA	*p* < 0.0001
k	Normal	Repeated-measures ANOVA	*p* = 0.014
l	Normal	Repeated-measures ANOVA	*p* = 0.01
m	Normal	Repeated-measures ANOVA	*p* = 0.63
n	Normal	Student’s *t* test	*p* = 0.64–0.92
o	Normal	ANOVA	*p* = 0.0009
p	Normal	ANOVA	*p* = 0.006
q	Normal	ANOVA	*p* < 0.0001
r	Normal	ANOVA	*p* = 0.03
s	Normal	ANOVA	*p* = 0.54–0.82
t	Normal	Pearson’s correlation coefficient	*p* = 0.03
u	Normal	Pearson’s correlation coefficient	*p* = 0.003
v	Normal	Pearson’s correlation coefficient	*p* = 0.01
w	Normal	Pearson’s correlation coefficient	*p* = 0.006
x	Normal	Pearson’s correlation coefficient	*p* = 0.019
y	Normal	Pearson’s correlation coefficient	*p* = 0.0007
z	Normal	Pearson’s correlation coefficient	*p* = 0.014
aa	Normal	Pearson’s correlation coefficient	*p* = 0.003
bb	Normal	ANOVA	*p* = 0.03
cc	Normal	ANOVA	*p* = 0.02
dd	Normal	ANOVA	*p* = 0.09-.16
ee	Normal	ANOVA	*p* = 0.03
ff	Normal	Student’s *t* test	*p* = 0.92–0.95
gg	Normal	ANOVA	*p* = 0.009
hh	Normal	ANOVA	*p* = 0.023
ii	Normal	ANOVA	*p* = 0.0003
jj	Normal	Pearson’s correlation coefficient	*p* = 0.047
kk	Normal	Pearson’s correlation coefficient	*p* = 0.035
ll	Normal	Pearson’s correlation coefficient	*p* = 0.0058
mm	Normal	Pearson’s correlation coefficient	*p* = 0.018
nn	Normal	Pearson’s correlation coefficient	*p* = 0.022

Study 1 mice underwent 3 d of reference memory place trials. There was an overall effect of age (*F*_(1,9)_ = 11.64, *p* = 0.007; data not shown; *b*) on place trials for reference memory, with the young showing lower cumulative proximities than the old. After the old cohort was subdivided into groups representing good reference memory (RG) and bad reference memory (RB), there was a significant main effect of Reference Memory Status in place (*F*_(2,8)_ = 17.90, *p* = 0.001; Fig. 1*B*; *c*) and probe trials (*F*_(2,8)_ = 19.53, *p* = 0.0008; Fig. 1*D*; *d*). Significantly lower proximities were evident in young, compared with RB for reference place trials (*p* = 0.0003; Fig. 1*B*) and compared with both RB and RG for probe trials (*p* = 0.0081–0.006; Fig. 1*D*), while RG had lower proximities than RB for reference memory place trials (*p* = 0.0002; Fig. 1*B*). Reversal place trials yielded no overall effect of Reference Memory Status (*F*_(2,8)_ = 1.49, *p* = 0.28; Fig 1*E*; *e*) in Study 1. There was, however, a significant Reference Memory Status effect in the reversal probe trial (*F*_(2,8)_ = 0.6.29, *p* = 0.0228; Fig. 1*F*; *f*), with RG exhibiting higher average proximity than young (*p* = 0.0097). There was a significant effect of Reference Memory Status on delayed matching-to-place task when examining naive and delay trials (*F*_(2,8)_ = 8.92, *p* = 0.0025; *g*), but no significant interaction between Status and Trial (*F*_(2,8)_ = 3.164, *p* = 0.0695; Fig. 1*H*; *h*), so no separate analysis was performed on individual trials. Significantly lower cumulative proximities were found in young compared with RG (*p* = 0.001) in the delayed matching-to-place task when naive and delay trials were averaged together. There was no significant effect of Reference Memory Status on cumulative proximity in the cued trials (*F*_(2,8)_ = 0.20, *p* = 0.82; Fig. 1*G*; *i*).

For Study 2 mice, there was a significant main effect of Reference Memory Status on average place trial performance for reference memory (*F*_(2,20)_ = 27.04, *p* < 0.0001; Fig. 1*C*; *j*). Young showed lower cumulative proximity than both RG (*p* = 0.006) and RB (*p* < 0.0001), and RG showed lower cumulative proximity than RB (*p* = 0.0004) in average place trials (Fig. 1*C*). There was a significant effect of Reference Memory Status on reversal place (*F*_(2,20)_ = 5.37, *p* = 0.014; Fig. 1*E*; *k*) and probe trials (*F*_(2,20)_ = 5.79, *p* = 0.01; Fig. 1*F*; *l*) in Study 2. RG had higher cumulative proximity than young in reversal place (*p* = 0.004; Fig. 1*E*) and probe trials (*p* = 0.003; Fig. 1*F*). There was no significant effect of Reference Memory Status on cumulative proximity in the cued trials (*F*_(2,20)_ = 0.48, *p* = 0.63; Fig. 1*G*; *m*).

### Protein palmitoylation status was related to cognitive declines

Recent data have indicated that old mice with good reference memory have higher levels of phosphorylation on tyrosine 1472 of the GluN2B subunit on synaptosomal detergent-resistant membrane (TxP) in the frontal cortex (Zamzow et al., 2016). We asked whether the increased p1472 observed in the TxP fraction of frontal cortex of old mice with good reference memory could be attributed to a change in the palmitoylation levels of GluN2B subunits. We used the acyl–biotin exchange method to precipitate acylated proteins from whole-cell lysates of the frontal cortex and hippocampus on Study 1 mice. Student’s *t* test was applied to the raw flotillin density data, and there were no significant differences between ages in the frontal cortex (*p* = 0.92; data not shown; *n*) or the hippocampus (*p* = 0.64; data not shown; *n*), so flotillin was used to correct for gel loading for the palmitoylation blots.

**Figure 2.**
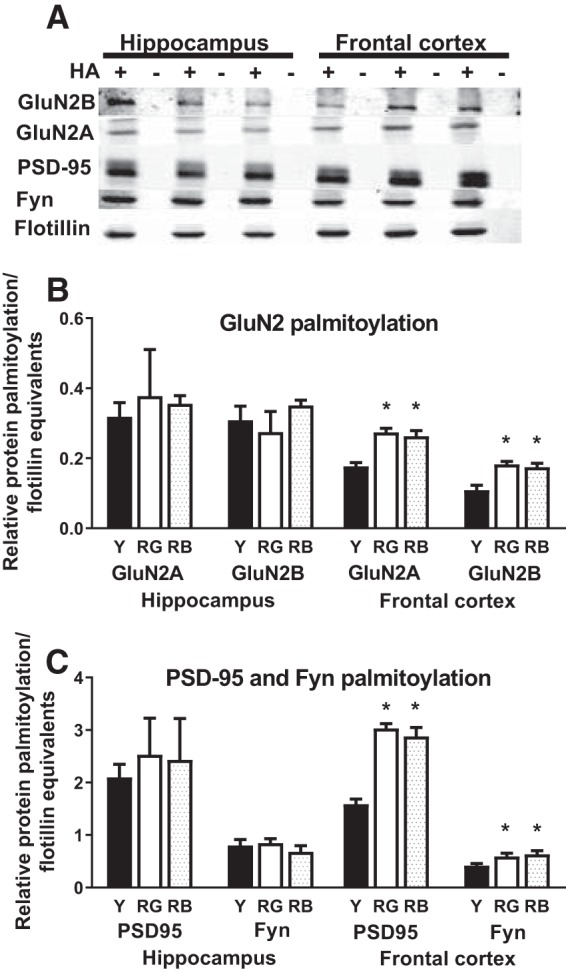
Protein palmitoylation. Age-related increase in palmitoylation in frontal cortex. ***A***, Representative blot of proteins precipitated by ABE. Order of lanes within each fraction are from left to right: young (+, − HA), RG (+, − HA), and RB (+, − HA) for each region. ***B***, ***C***, There was an age-related increase in palmitoylation in the frontal cortex for GluN2 subunits (***B***), as well as, PSD-95 and Fyn (***C***). *p < 0.05 for difference from young (ANOVA and Fisher’s PLSD). *N* = 3–5. Data are the mean ± SEM. HA, Hydroxylamine treated.

The GluN2A (*F*_(2,8)_ = 19.05, *p* = 0.0009; *o*) and GluN2B (*F*_(2,8)_ = 10.26, *p* = 0.006; Fig. 2*A*,*B*; *p*) subunits had significant Reference Memory Status effects on palmitoylation in frontal cortex lysates. PSD-95 (*F*_(2,8)_ = 50.66, *p* < 0.0001; *q*) and Fyn (*F*_(2,8)_ = 5.21, *p* = 0.03; Fig. 2*A*,*C*; *r*) also exhibited significant reference memory status effects in the frontal cortex. In each case though, both the RG and RB had higher levels of palmitoylation than the young (*p* < 0.001 to 0.05; Fig. 2). Interestingly, there was no significant Reference Memory Status effect on the relative palmitoylation levels of proteins in the hippocampus (*p* = 0.54-0.82; Fig. 2; *s*).

**Figure 3.**
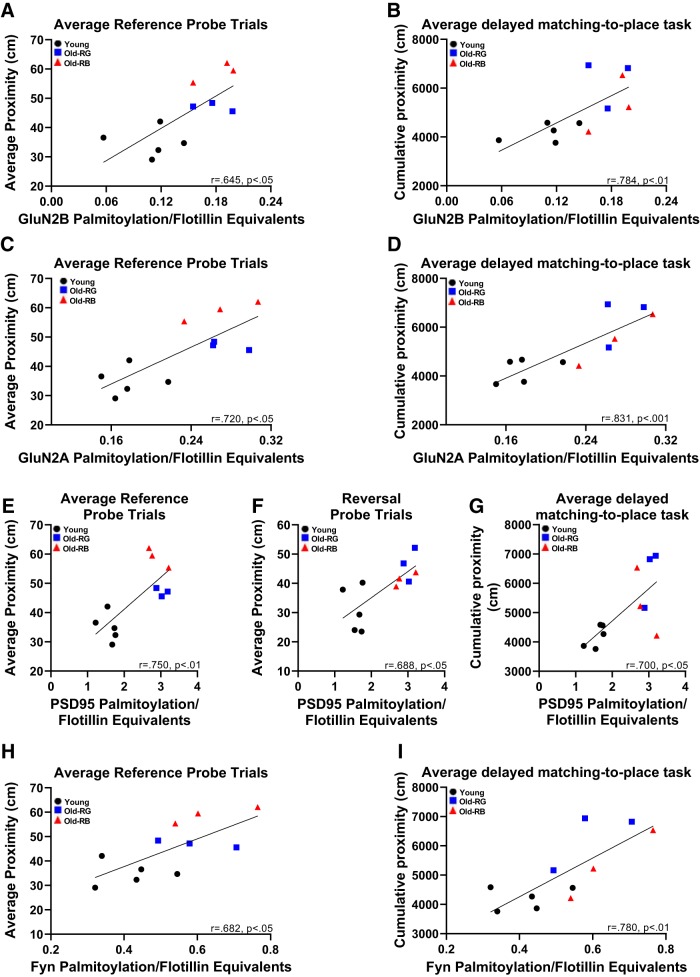
Protein palmitoylation correlated with behavioral data. ***A–I***, Significant correlations were found between greater levels of palmitoylation, and deficits in performance for reference probe trials and in the averaged delayed matching-to-place task for GluN2B (***A*** and ***B***, respectively), GluN2A (***C*** and ***D***), and Fyn (***H*** and ***I***). ***E–G***, Palmitoylation of PSD-95 correlated with reference probe trials (***E***), reversal probe trials (***F***), and delayed matching-to-place task (***G***). *N* = 3-5. *r*, Pearson correlation coefficient.

Pearson’s correlation coefficients were calculated for protein palmitoylation status and behavioral measures. When learning scores were averaged across trials, it was found that higher levels of palmitoylation of Glun2A, GluN2B, PSD-95, and Fyn all correlated with poorer scores on some of the behavioral tests. The palmitoylation status of GluN2B significantly correlated with both reference probe trial performance (*p* = 0.03; Fig. 3*A*; *t*) and averaged delayed matching-to-place task performance (*p* = 0.003; Fig. 3*B*; *u*), but not reference memory or reversal place, or reversal probe trials (*p* = 0.15-.22; data not shown). Similar results were revealed when the correlation between the palmitoylation status of GluN2A, PSD-95, and Fyn, and learning scores were determined. The palmitoylation status of Glun2A (*p* = 0.01; Fig. 3*C*; *v*), PSD-95 (*p* = 0.006; Fig. 3*E*; *w*), and Fyn (*p* = 0.019; Fig. 3*H*; *x*) all correlated to reference probe trial performance. Additionally, GluN2A (*p* = 0.0007; Fig. 3*D*; *y*), PSD-95 (*p* = 0.014; Fig. 3*F*; *z*), and Fyn (*p* = 0.003; Fig. 3*I*; *aa*) correlated with averaged delayed matching-to-place task performance. Further analysis of the delayed matching-to-place task showed palmitoylated GluN2B (*r* = 0.80; *p* = 0.003), GluN2A (*r* = 0.81; *p* = 0.003), PSD95 (*p* = 0.03), and Fyn (*r* = 0.73; *p* = 0.01) significantly correlated with the naive trial performance, while only GluN2A (*r* = 0.64; *p* = 0.03) and Fyn (*r* = 0.61; *p* = 0.045) showed relationships to the delay trial (data not shown). Average delayed matching-to-place task performance showed a nearly significant relationship to both types of reversal trials (*r* = 0.54; *p* = 0.08–0.09; data not shown). The palmitoylation status of PSD-95 also correlated with reversal probe performance (*p* = 0.017; Fig. 3*F*). There was no significant correlation of the palmitoylation status of GluN2A, PSD-95, or Fyn to reference (*p* = 0.0569–0.1179; data not shown) or reversal place trial performances (*p* = 0.091–0.33; data not shown). Palmitoylation status was also not significantly correlated to reversal probe performance for either GluN2A or Fyn (*p* = 0.068–0.28; data not shown).

### Fatty acid transporters reduced with increased age

**Figure 4.**
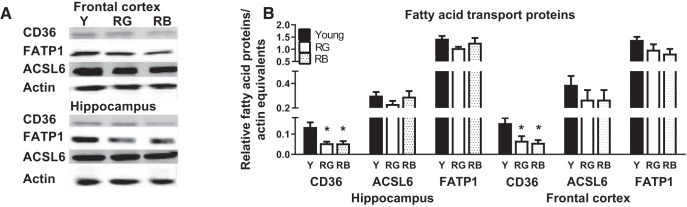
Fatty acid transport proteins. ***A***, Fatty acid transport proteins showed reductions with age. Representative blot of proteins. ***B***, CD36 expression decreased within both RG and RB. FATP1 was decreased between young and all old in the frontal cortex (*p* = 0.03), but was not significantly affected by reference memory status. *p < 0.05 for difference from young (ANOVA and Fisher’s PLSD). *N* = 3–5. Data are the mean ± SEM.

Up-regulation of fatty acid transport proteins might lead to increased palmitoyl-CoA, thereby driving an increase in protein palmitoylation. We, therefore, examined expression levels of fatty acid transport proteins, CD36 and FATP1, and the palmitate esterification protein ACSL6 in whole-cell lysates from the frontal cortex and hippocampus in Study 1 mice. There was a significant effect of Reference Memory Status on CD36 in the frontal cortex (*F*_(2,8)_ = 5.53, *p* = 0.03; *bb*) and hippocampus (*F*_(2,8)_ = 5.77, *p* = 0.02; Fig. 4*A*,*B*; *cc*), but both RG (*p* = 0.02–0.3) and RB (*p* = 0.02) had lower CD36 expression than young in both regions. There was also a significant age-related reduction in FATP1 in the frontal cortex (*F*_(1,9)_ = 6.37, *p* = 0.03; data not shown). There was no significant Reference Memory Status effect in the frontal cortex or hippocampus on the expression of ACSL6 (*p* = 0.36–0.44) or FATP1 (*p* = 0.09–0.16; Fig 4*A*,*B*; *dd*). These data suggest that increased protein palmitoylation levels in the frontal cortex occurred despite reductions in fatty acid transporter expression with increased age.

### APT1 increased palmitoylation with age

**Figure 5.**
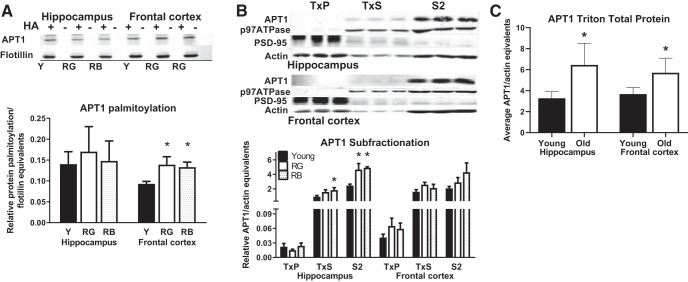
APT1 palmitoylation and localization. ***A***, Representative blot: order of lanes from left to right are young, RG, and RB. ***A***, Palmitoylation of APT1 increased with age in frontal cortex, but not in the hippocampus from Study 1. ***B***, APT1 expression increased in the TxS and S2 fractions from hippocampus of old mice from Study 2. ***C***, Total APT1 protein increased with age in the frontal cortex and hippocampus. *p < 0.05 for difference from young (ANOVA and Fisher’s PLSD). *N* = 3–11. Data are the mean ± SEM. F, Frontal cortex; H, hippocampus.

We next examined the subcellular localization and palmitoylation status of APT1 because of the broad-range ability to control its own palmitoylation as well as depalmitoylation of other proteins. We found a significant Reference Memory Status effect on the palmitoylation status of APT1 in the frontal cortex (*F*_(2,8)_ = 5.54, *p* = 0.03; Fig. 5*A*; *ee*), but not the hippocampus (*p* = 0.88) in Study 1. Both RG (*p* = 0.02) and RB (*p* = 0.03) had higher palmitoylated APT1 expression than young in the frontal cortex (Fig. 5*A*). We next examined the cellular localization of APT1 using subfractionated lysates from a previous study (Study 2) investigating age-related changes in subcellular localization of NMDA receptors and their effector proteins (Zamzow et al., 2016). Student’s *t* test was applied to the raw actin density data, and there were no significant differences between ages in the frontal cortex (*p* = 0.95; data not shown; *ff*) or the hippocampus (*p* = 0.92; data not shown; *ff*), so actin was used to correct for gel loading for the Triton cellular fractionation blots.

Although there was no effect of Reference Memory Status on the cellular fractions from the frontal cortex (*p* = 0.13–0.26; Fig. 5*B*), there was a trend for age-related increase in APT1 expression in the TxS and S2 fractions (*p* = 0.07). There was a significant effect on the TxS (*F*_(2,20)_ = 3.84, *p* = 0.03) and S2 (*F*_(2,17)_ = 6.24, *p* = 0.009; Fig. 5*B*; *gg*) fractions from the hippocampus, but no effect was found in the TxP fraction (*p* = 0.58). RB had significantly higher APT1 in the hippocampal TxS (*p* = 0.02) fraction than young and both RB (*p* = 0.007) and RG (*p* = 0.01) had higher levels in the hippocampal S2 fraction than young (Fig. 5*B*). The total APT1 protein in all fractions was averaged, and a significant age-related increase in expression was found in the frontal cortex (*F*_(1,9)_ = 7.43, *p* = 0.023; Fig. 5*C*; *hh*) and in the hippocampus (*F*_(1,18)_ = 19.73, *p* = 0.0003; Fig. 5*C*; *ii*).

### APT1 palmitoylation status correlated with memory and protein palmitoylation

**Figure 6.**
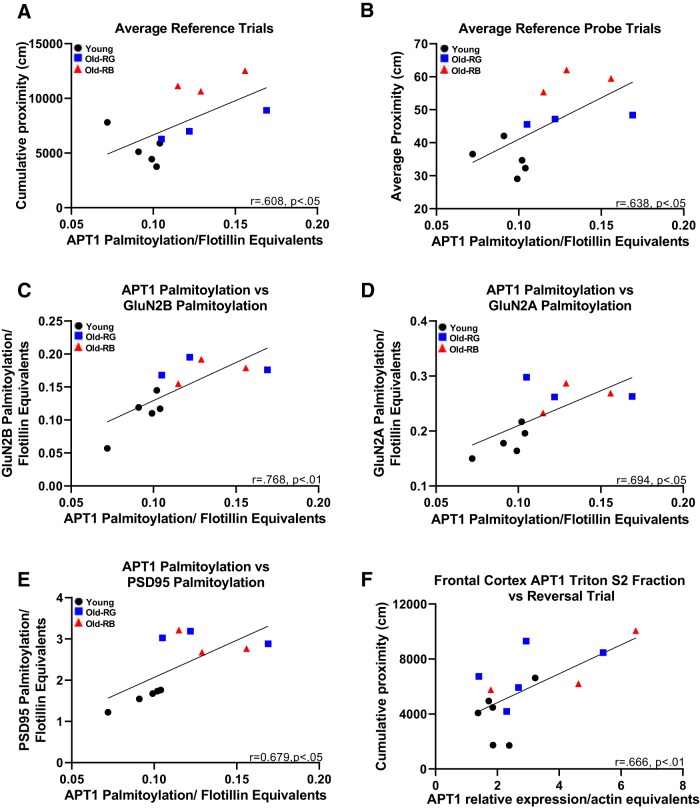
APT1 correlated with spatial memory performance. *A*, ***B***, An increase in APT1 palmitoylation correlated with poorer memory scores for reference (***A***) and probe (***B***) trials. ***C–E***, APT1 palmitoylation status correlated with the palmitoylation status of GluN2B (***C***), GluN2A (***D***), and PSD-95 (***E***). ***F***, Higher levels of APT1 in the S2 fraction of frontal cortices correlated with poorer cognitive flexibility. *N* = 3–6. *r*, Pearson correlation coefficient.

The mode of action of APT1 is to depalmitoylate palmitoylated proteins on the plasma membrane so that they can be recycled back to the Golgi apparatus to be repalmitoylated and return to the plasma membrane (Kong et al., 2013). Therefore, we expected that the correlation between the palmitoylation status of APT1 and memory performance would be similar to the relationships shown by palmitoylated GluN2A, GluN2B, Fyn, and PSD-95. However, that was not the case. Palmitoylated APT1 correlated with both reference place (*p* = 0.047; Fig. 6*A*; *jj*) and reference probe (*p* = 0.035; Fig. 6*B*; *kk*) trials across aging, but, unlike the aforementioned proteins, there was no correlation to delayed matching-to-place task or reversal trials (*p* = 0.066–0.216). Despite the palmitoylation status of APT1 not correlating with the same behavioral assays as the other proteins examined, we found a remarkably consistent correlation between the palmitoylation status of APT1 and GluN2A (*p* = 0.0058; Fig. 6*C*; *ll*), GluN2B (*p* = 0.018; Fig. 6*D*; *mm*), and PSD-95 (*p* = 0.022; Fig. 6*E*; *nn*). Interestingly, there was no significant correlation between APT1 palmitoylation and Fyn palmitoylation (*p* = 0.24; data not shown). The subfractionation data were also subjected to correlational analysis for both the hippocampus and the frontal cortex. The S2 fraction of the frontal cortex significantly correlated with reversal trials (*p* = 0.009; Fig. 6*F*), while none of the other fractions correlated with any of the memory data (*p* = 0.08–0.91).

## Discussion

In this study, young and old mice were behaviorally characterized with the Morris water maze. Old mice were separated into two groups based on poor or good reference memory performance, as a follow-up to a previous study (Zamzow et al., 2016). Old mice that performed well in spatial memory trials suffered from cognitive flexibility and delayed matching-to-place task deficits. Palmitoylation levels of several NMDAR complex proteins increased in an age-dependent manner and correlated to delayed matching-to-place task and reference memory probe scores, but showed no relationship to performance levels in the reference memory place trials. Finally, APT1 showed increased palmitoylation with age in the frontal cortex and increased expression in the TxS and S2 fractions from the hippocampus. Unlike the NMDAR complex proteins, the palmitoylation status of APT1 correlated with reference memory and not the delayed matching-to-place task.

The Morris water maze is a tool that has a long track record of assessing many aspects of spatial memory in a variety of species, including spatial reference, reversal, and short-term or working memory (Vorhees and Williams, 2006; Paul et al., 2009). While several brain regions coordinate memory acquisition, consolidation, and retrieval, evidence suggests that the prefrontal cortex is primarily involved with executive functions, including short-term or working memory and cognitive flexibility (reversals; Drag and Bieliauskas, 2010; Euston et al., 2012; Funahashi, 2017). The 10 min delay trial in our delayed matching-to-place task is unlikely to reflect working memory, as defined by focusing attention over a shorter time period (Engle, 2018). It may involve longer-term memory consolidation (Steele and Morris, 1999), but it likely decays, since the information is only beneficial for one session, so it could be referred to as short-term memory (Cowan, 2008). The naive trial results may be influenced by the search strategy and/or cognitive flexibility of the animal (Williams et al., 2003; Vorhees and Williams, 2006). Performance averaged across naive and delay trials thus likely reflected multiple executive functions. There is evidence that this type of matching-to-place task involves NMDA receptors in the prefrontal cortex and hippocampus (Steele and Morris, 1999; Das and Magnusson, 2008).

In agreement with previous work, our study found that old mice with good reference memory suffered from deficits in cognitive flexibility (Zamzow et al., 2016). In addition, we found that old mice that performed poorly on the Morris water maze spatial reference task performed better than their age-matched counterparts on the reversal cognitive flexibility task, and overall in the delayed matching-to-place task. Our data, however, only partially agreed with other reports that found an age-related difference in spatial memory as assessed by the Morris water maze, but group-specific differences in old rodents in cognitive flexibility testing (Barense et al., 2002; Schoenbaum et al., 2002), and one report (Beas et al., 2013) that found a disagreement between short-term memory and cognitive flexibility scores in old rodents. These studies differed from ours in several key ways. First, they all used rats as a rodent model, while we used C57BL/6 mice. Second, we used the Morris water maze to test spatial reference memory, cognitive flexibility, and delayed matching-to-place task, while the other studies used the Morris water maze only for the spatial reference portion of their studies. Third, the previously reported short-term memory task used delays of <25 s and interpreted this as reflecting working memory (Beas et al., 2013). Together, our data seem to support a theory of multiple compensatory mechanisms used by old mice in memory trials, which may conflict with other cognitive functions (i.e., reference memory vs executive functions).

Several key proteins in the NMDAR complex showed increased palmitoylation in the frontal cortices of old mice. Previous evidence suggested that higher levels of palmitoylation in the prefrontal cortex may act as a compensatory mechanism in old mice with good reference memory. Palmitoylation of PSD-95 clusters the protein on synaptic membranes (El-Husseini et al., 2002). However, data indicates that mutating the N-terminal cysteine residues responsible for the palmitoylation of PSD-95 does not eliminate association with the synaptic membrane (Topinka and Bredt, 1998). In normal hippocampal neurons, the half-life of palmitate on PSD-95 is ∼2 h, but the half-life of the PSD-95 protein itself is 30 h (El-Husseini et al., 2002). This indicates dynamic cycling of PSD-95 on and off synaptic membranes. The increased palmitoylation of PSD-95 in the frontal cortex of aged mice may indicate a perturbation in the palmitoylation cycle, but not a change in localization.

The palmitoylation of NMDAR complex proteins in the present study showed an increase with age, but did not show a relationship to reference learning within the old mice. Because palmitoylation is essential for phosphorylation of the GluN2B subunit (Hayashi et al., 2009; Trepanier et al., 2012), higher levels of palmitoylation did not appear to explain the previous findings of greater phosphorylation at tyrosine 1472 of GluN2B subunits in better old reference learners or alterations in Fyn expression in poor old learners (Zamzow et al., 2016). We thus explored for relationships with other behavioral measures across aging with the use of correlational analysis. The strongest correlations were found between palmitoylated GluN2B, GluN2A, and Fyn, and averaged performance in the delayed matching-to-place task. This appeared to be due to a relationship to the naive trial measures for each of these proteins and the delayed trial for GluN2A and Fyn. All three of these palmitoylated proteins also showed a relationship with the reference probe trials across aging. This suggests that palmitoylation of GluN2B, GluN2A, and/or Fyn in the frontal cortex interfered with executive functions, such as cognitive flexibility and/or search strategy in naive trials (Williams et al., 2003; Vorhees and Williams, 2006) and short-term memory or rapid consolidation of escape location information in the delayed trials (Steele and Morris, 1999); and spatial reference memory in probe trials. Palmitoylated PSD-95 showed relationships to reference and reversal probe trials and the delayed matching-to-place task, but the clustering of the young and aged suggested that this may just reflect unrelated aging changes in both measures.

In accordance with a previous study, we found that the GluN2B and GluN2A subunits, along with the Src kinase Fyn and the synaptic scaffolding protein PSD-95, were all palmitoylated in greater numbers in the frontal cortices of old mice (Zamzow et al., 2014). In that study, we also examined levels of free palmitate and palmitoyl-CoA to determine whether more substrate for protein palmitoylation was available in the frontal cortices of old mice. Although no age-related differences were detected in free palmitate or palmitoyl-CoA, the assay we used did not include palmitate bound to proteins. Approximately 50% of the cellular acyl-CoA pool is bound to proteins (Juguelin et al., 1991). Considering that there was significantly more cellular palmitate and palmitoyl-CoA present in the frontal cortices than the hippocampi of all mice in that study and our current results indicated an increased number of palmitoylated proteins in the frontal cortices of old mice, we sought to examine possible mechanisms that might lead to an overall increase in the palmitoyl cellular pool. There was a significant age-related decrease in the fatty acid transport protein CD36 in both the frontal cortex and hippocampus, and FATP1 in the frontal cortex. This would indicate that the higher levels of protein palmitoylation in the frontal cortices of old mice could not be contributed to greater expression of fatty acid transport proteins. However, FATP1 and ACSL1 can bind together to enhance esterification of fatty acids (Anderson and Stahl, 2013), and there are two alternatively spliced isoforms of ACSL6 (Van Horn et al., 2005). It is not known whether FATP1 and ACSL6 interact or whether ACSL6 is alternatively spliced in aged mice, but many brain mRNAs become alternatively spliced with age (Tollervey et al., 2011), and an age-related shift in ACSL6 isoform expression might have a greater affinity for FATP1, thereby altering the kinetics of palmitate esterification. Whatever the mechanism, protein expression of fatty acid transport proteins does not seem to be a viable explanation for the rise in esterified palmitate in the frontal cortices of aged mice.

The palmitoylation cycle in cells is conducted by PATs and depalmitoylated by APTs (Linder and Deschenes, 2007). The family of PATs consists of 25 proteins known as DHHC, for the aspartate-histidine-histidine-cysteine motif they all share, while only 2 APT proteins are known to exist (Linder and Deschenes, 2007; Greaves and Chamberlain, 2011). The DHHC proteins are highly degenerate, and several different isoforms will palmitoylate a single protein. The PATs DHHC2, 3, 7, 8, and 15 palmitoylate PSD-95, DHHC3 palmitoylates GluN2A and GluN2B, and DHHC2, 7, 15, 20, and 21 palmitoylate Fyn; therefore, it seems unlikely that an alteration in PAT expression would cause such widespread changes in protein palmitoylation in the frontal cortices of aged mice. Our examination of APT1 yielded some interesting results. We found that, like GluN2A and GluN2B, Fyn, and PSD-95, greater numbers of APT1 were palmitoylated in the frontal cortices, but not in the hippocampi, of aged mice. However, there was disagreement between the APT1 and the other proteins when it came to memory data. One explanation is that APT1 is not directly involved in depalmitoylating some or all of the proteins. Indeed, as many as 21 other thioesterase enzymes may have activity against palmitoylated proteins (Yokoi et al., 2016). The authors found that PSD-95 is depalmitoylated by the thioesterase ABHD17; however, this enzyme had only limited activity against palmitoylated GluN2A, meaning its palmitoylation status is governed by one or more other thioesterases. New evidence implicates PPT1 as a thioesterase that depalmitoylates both GluN2B and Fyn. Interestingly, the PPT1 knock-out mice in the study had greater susceptibility to excitotoxicity, which was reversed by palmitoylation inhibitors (Koster et al., 2018). If APT1 does not target GluN2B, Fyn, or PSD-95, this suggests that a global perturbation in the palmitoylation cycle, not a defect in APT1 activity, may be causing the uptick in the percentage of proteins that are palmitoylated in the aged frontal cortex.

To date, the ability to autodepalmitoylate has only been characterized in APT1. The thioesterases APT1 and APT2 undergo a unique palmitoylation/depalmitoylation cycle that regulates the activity of each enzyme (Kong et al., 2013). Depalmitoylation of both APT1 and APT2 is conducted by APT1, allowing both enzymes to be localized near their substrates and recycle back to the Golgi apparatus when depalmitoylated. The autodepalmitoylating activity of APT1 modulates the cellular localization of the enzyme, acting like a gatekeeper by residing on the Golgi apparatus to control excess palmitoylation of proteins, including APT1 (Vartak et al., 2014). Vartak et al. (2014) reported that APT1 will normally be enriched on Golgi membranes over the cytosol. An age-related increase in palmitoylated APT1 suggests that the protein is kinetically trapped on the Golgi apparatus. Considering this, we looked at subcellular localization of APT1 in lysates from a previous experiment. Because our method of subcellular subfractionation was unable to separate light endomembranes from the cytosolic fraction, we could not ascertain whether there was an age-related shift toward endomembranes (Zamzow et al., 2016). However, increased palmitoylation of APT1 would indicate that dynamic shuttling on and off Golgi membranes may be diminished with age in the frontal cortex. This theory fits well with the correlational data between APT1 palmitoylation and palmitoylation levels of GluN2A, GluN2B, Fyn, and PSD-95. APT1 palmitoylation associated well with palmitoylation of GluN2A, GluN2B, and PSD-95, but not Fyn. APT1, GluN2A, GluN2B, and PSD-95 are all palmitoylated by a PAT that resides on the Golgi apparatus (Hayashi et al., 2009; Noritake et al., 2009; Vartak et al., 2014). Conversely, Fyn is shuttled to the synaptic membrane with Rab11 and seems to avoid the Golgi apparatus during exocytosis (Sato et al., 2009). This would suggest that the age-related disturbance of the palmitoylation cycle in the frontal cortex can disrupt the trafficking and localization of each protein in a unique way.

We found that the overall protein expression of APT1 was higher in the hippocampi and frontal cortices of old mice. This is in good agreement with the results of a previous study (Tatro et al., 2013) that found that APT1 expression was higher in the hippocampi of old mice due to a loss of miR138 with age, but was not associated with working memory assessed by the novel object recognition task. The single-trial object recognition test used by Tatro et al. (2013) is hippocampal dependent and they did not assess cognitive flexibility, so it is not known whether the prefrontal cortex was a factor in their findings. On the other hand, we assessed aspects of spatial memory that are attributed to both the prefrontal cortex and the hippocampus. The evidence from this study indicates that two mechanisms may be in play regarding APT1 in the aged brain. The first is an increased level of palmitoylation, most probably due to a disturbed cellular palmitoylation cycle, that has detrimental effects on reference memory, possibly through action on other proteins. For example, palmitoylated GAP-43 is a substrate for APT1, and GAP-43 is important for spatial memory storage (Holahan et al., 2007). The second mechanism is evidenced by the correlation data that showed poorer cognitive flexibility with increased concentration of APT1 in the S2 fraction from frontal cortices. Although APT1 expression increased with age in both the frontal cortices and hippocampi of mice, cognitive flexibility was associated only with frontal cortex expression. This may be due to a buildup of cytosolic APT1, even as more APT1 accumulates on the Golgi. Vartak et al. (2014) found that APT1 was still active when it was depalmitoylated and soluble. It is conceivable that an increase in cytosolic APT1 can modulate the palmitoylation levels of some proteins on the plasma membrane, affecting cognitive flexibility. Simultaneously, accumulation of palmitoylated APT1 on endomembranes would represent a disturbance in the balance of the recycling rhythm of the neuron, causing poorer performance during reference memory trials.

In this study, we found that old mice that scored poorly on spatial reference memory tests had better cognitive flexibility and overall performance in a delayed matching-to-place task than those who learned an initial location well. Several NMDAR- and receptor-associated proteins, as well as the depalmitoylating enzyme APT1, had higher levels of palmitoylation in the frontal cortices of old mice, but not the hippocampus. This occurred despite reductions in fatty acid transport proteins in both regions. The increases in palmitoylation were associated with reduced abilities in reference memory and executive functioning across aging, but did not account for cognitive differences between old mice. While APT1 palmitoylation was associated with deficits in reference memory, the increased palmitoylation of the enzyme could not account for all of the consequences of higher levels of palmitoylation of the NMDAR complex proteins studied. Further work should elucidate the causes and consequences of a disturbed palmitoylation cycle in the aging brain.

## Synthesis

Reviewing Editor: Mark Baxter, Mount Sinai School of Medicine Decisions are customarily a result of the Reviewing Editor and the peer reviewers coming together and discussing their recommendations until a consensus is reached. When revisions are invited, a fact-based synthesis statement explaining their decision and outlining what is needed to prepare a revision will be listed below. The following reviewer(s) agreed to reveal their identity: Sarah Johnson, J. Leigh Leasure.

Dear Dr. Zamzow,

Two reviewers and I have reviewed your manuscript, “The protein palmitoylation cycle is perturbed in the frontal cortex, but not the hippocampus of aged mice”. An integrative summary of our comments follows

Our opinions differ somewhat on the paper. I think it is difficult to tell what the unique, new contributions of this study are, given that a previous paper reported differences in palmitoylation between young and aged mice in frontal cortex and hippocampus. It appears that this paper attempted to explore some possible biochemical mechanisms and also to relate this to differences in behavior between young and aged mice, although it was not possible to find a link to individual differences in behavior even though some aged mice performed within the range of the young mice. This would seem to belie the conclusion “a contribution to age-related memory declines cannot be ruled out, since young performed better than both older groups for reference memory” (p. 13), although without correlational analyses it is difficult to understand what the relationship between biochemistry and behavior is. The manuscript would benefit from clarifying what the new, distinct contributions are - we do not expect every paper to contain a major advance, but it needs to be clear what the advance is.

The behavioral data could be presented in a more straightforward and hypothesis-driven way. The relationship between reference memory performance and the working memory and reversal tasks has been reported before, and the new data behavioral data in this paper (the Study 2 cohort) appear to replicate this pattern of performance, at least for the reversal task since working memory was not tested in Study 2. To the extent that the major behavioral classification seems to be based on performance in the reference memory task, it may be better to describe the behavioral data from both studies in one figure, and then turn to the biochemical analyses. Given that reversal performance in the aged mice is dissociated from reference memory performance, it may be informative to conduct correlational analyses with reversal performance as well. In the absence of such analyses, it is not obvious that reporting the working memory/reversal data adds anything to the paper. Was swim speed analyzed during the reference memory trials? This could be reported as an additional control comparison, complementing the cued trials.

The statistical analyses require some clarification. It is not clear from the methods how statistical analyses were implemented. In many cases, e.g. for behavioral variables and contrast of biochemical measures in frontal cortex versus hippocampus, a repeated measures within subjects design would be appropriate. If this approach was not taken, data could be re-analyzed to clarify differences in expression patterns between the two brain regions. It is also not clear how conclusions were drawn relating the reference memory status ('good' vs 'bad') to biochemical measures. While the text of the results frequently reports significant main effects of reference memory status, inspection of the figures suggests that for all biochemical data, these main effects are driven by both aged groups showing different expression than young. Correlation or regression analyses should be used to more directly assess if behavioral performance was related to palmitoylation measures. Indeed, this is one of the reasons to use an average proximity score, to provide a single measure that can be related to biochemical measures in correlational analyses. More detail must also be provided, including list of between and within subject factors entered in ANOVAs and alpha level.

Throughout the manuscript there are what appear to be placeholders for citations (“Add Author”); the appropriate citations should be included.

Minor comments -

Introduction - The sentence 'Short-term working memory declines with age, but so does more complex memory' should be tempered. While it may be assessed on a shorter timescale, this doesn't preclude working memory from being complex.

You should comment on the use of “working memory” in this context, which seems to refer to the Olton “working/reference” distinction, rather than the more common use of “working memory” in cogntiive psychology that pertains to information held in the focus of attention for short time intervals. A 10-minute delay would vastly exceed working memory capacity, in the common sense of the term.

Methods: More detail should be provided on the dissection method of frontal cortical regions and hippocampus, particularly given the divergent findings from these brain regions.

Catalog / product numbers and lot numbers should be provided for antibodies used in Western blots (Table 3.1).

It might be of interest to return the 3 'bad' performing young mice into the data set for correlation or regression analyses.

Raw density values for both palmitoylated Flotillin and actin should be included and analyzed to confirm no differences were observed across experimental groups.

Results: Figure 4: It would be helpful to redefine the abbreviations for subcellular fractions in the legend, as well as meaning of 'F' and 'H'. There may also be a typo In the description for panel E- results suggest these data are from Study 2 but legend indicates from Study 1.

## Author Response

Dr. Mark Baxter

Reviewing Editor

eNeuro

Dear Dr. Baxter:

We are submitting our revised manuscript entitled “Higher levels of protein palmitoylation in the frontal cortex across aging were associated with reference memory and executive function declines” for consideration for publication in eNeuro.

We have done our best to address the concerns of the reviewers, including the addition of clarity and correlational data. We have detailed the changes below in the Response to Reviewers and have indicated the changes within the manuscript with yellow highlight. We hope that you will now find this manuscript appropriate for publication in eNeuro.

Thank you for your time and consideration.

Response to Reviewers: Changes are indicated in the manuscript by yellow highlight

1. Contribution

a. After correlational analysis, we are now able to show an association between increased palmitoylation of NMDAr complex proteins and poorer performance in the delayed matching-to-place task and reference memory across ages (see below). We have also clarified the discussion to demonstrate how APT1 palmitoylation serves as a proxy for the state of the palmitoylation cycle of the cell as a whole. We believe these two pieces of information are significant contributions to the discipline.

2. Behavioral data in one figure

a. We have condensed all of the behavioral data from both studies into Figure 1.

3. Swim speed

a. Swim speed is likely to be slower in the aged animals, based on our previous results (Magnusson KR: Mech Ageing Dev 1998, 104:227-248), so would not complement the results of the cued trials as a control. We chose to use the proximity measures, corrected for start position and average swim speed, to reduce the effects of swim speed.

4. Clarification of statistical analysis

a. We have included the fact that repeated measures ANOVA was used for the behavior.

b. We have included the factors included in ANOVAs and alpha level.

5. Correlational analysis

a. We performed correlational analysis between the palmitoylation status of each protein and behavioral performance. This data was included in Figures 3 and 6.

b. We performed correlational analysis between APT1 palmitoylation and palmitoylation of each of the remaining proteins. This data was included in Figure 6.

c. We performed correlational analysis between total protein levels of APT1 in all cellular subfractions in Study 2. This data was included in Figure 6.

6. Working memory

a. We have more correctly referred to this task as a delayed matching-to-place task and discussed what the naive and delayed trials may reflect, concluding that the averaged performance over naive and delayed trials likely includes multiple executive functions.

7. Dissection details

a. We have added information on the dissection method.

8. Catalog/product numbers.

a. We have included product numbers. We do not have the lot number information, so those are not included.

9. 3 bad performing young mice.

a. No protein work was performed on the poor performing young mice.

10. Raw values analyzed for actin and flotillin

a. A Student's t-test was performed on the raw data for actin and flotillin. P values were added to the manuscript.

11. Figure 4 legend.

a. We have clarified the abbreviations in Figure 4 legend.

b. We have corrected the reference for panel E.
